# Corrigendum: Expedited Holonomic Quantum Computation via Net Zero-Energy-Cost Control in Decoherence-Free Subspace

**DOI:** 10.1038/srep44957

**Published:** 2017-03-23

**Authors:** P. V. Pyshkin, Da-Wei Luo, Jun Jing, J. Q. You, Lian-Ao Wu

Scientific Reports 6: Article number: 3778110.1038/srep37781; published online: 11 25 2016; updated: 03 23 2017

In the original version of this Article, Affiliations 1, 2 and 3 were not listed in the correct order. The correct affiliations are listed below:

Affiliation 1

Beijing Computational Science Research Center, Beijing 100084, China.

Affiliation 2

Department of Theoretical Physics and History of Science, The Basque Country University (EHU/UPV), PO Box 644, 48080 Bilbao, Spain.

Affiliation 3

Ikerbasque, Basque Foundation for Science, 48011 Bilbao, Spain.

This error has now been corrected in the PDF and HTML versions of the Article.

